# Alteration of Pressure-Induced Vasodilation in Aging and Diabetes, a Neuro-Vascular Damage

**DOI:** 10.3389/fphys.2019.00862

**Published:** 2019-07-03

**Authors:** Maxime Fouchard, Laurent Misery, Raphaële Le Garrec, Dominique Sigaudo-Roussel, Bérengère Fromy

**Affiliations:** ^1^LIEN, F-29200, University of Brest, Brest, France; ^2^Department of Dermatology, University Hospital of Brest, Brest, France; ^3^UMR 5305 CNRS, University of Lyon 1, Lyon, France

**Keywords:** pressure-induced vasodilation, low pressure, skin blood flow, pressure ulcer, diabetes, aging

## Abstract

Skin is constantly subjected to pressure at different levels. Pressure-induced vasodilation (PIV) is one of the response mechanisms to low pressure that maintains the homeostasis of the skin. PIV results from the interaction of primary afferent nerves and vascular endothelium of skin vessels. Thanks to this cutaneous neuro-vascular interaction, the cutaneous blood flow increase allows the maintenance of an optimal level of oxygenation and minimizes the lack of vascularization of the skin tissue under low pressure. It seems to be associated with the cutaneous protection mechanisms to prevent pressure ulcers. In some contexts, where microangiopathy and neuropathy can occur, such as aging and diabetes, PIV is impaired, leading to a dramatic early decrease in local skin blood flow when low pressure is applied. In aging, PIV alteration is due to endothelial dysfunction, essentially from an alteration of the nitric oxide pathway. In the inflamm-aging context, oxidative stress increases leading to endothelial cell and nerve damages. An age-related sensory neuropathy will exacerbate the alteration of PIV during the aging process. In diabetes, non-controlled hyperglycaemia leads to an increase in several pathological biochemical pathways that involve oxidative stress and can affect PIV. Sorbinil, alagebrium and alpha-lipoic acid are able individually to restore PIV through a possible oxidative stress reduction. Candesartan, an angiotensin II type 1 receptor blocker, is also able to restore PIV and prevent pressure ulcer formation. The possibility of preventing pressure ulcer associated to diabetes and/or aging with the restoration of PIV seems to be a promising research path.

## Introduction

The skin, nervous system and immune system are not independent systems but are closely associated and use the same language of cytokines and neurotransmitters in the context of the neuroimmunocutaneous system (Misery, 1997).

Among the numerous interactions within the neuroimmunocutaneous system, those that take place between the blood vessels and the nervous system are probably some of the first studied and are increasingly understood (Kodji et al., 2016). Pathological conditions, including diabetes (Vinik et al., 2001), small-fiber neuropathies (Misery et al., 2013), chronic wounds (Chéret et al., 2014), skin inflammation (Gouin et al., 2015) and the use of toxic substances such as nicotine (Misery, 2004), provide interesting data explaining the physiological mechanisms of neurovascular interactions.

The human skin is subjected to different ranges of pressure all the time, but adaptive mechanisms help it to remain intact. When low pressure ( < 20 kPa) is applied to healthy skin, local vasodilation appears. It allows an increase in the skin blood flow and helps keep an optimal level of oxygenation in the skin even with pressure on it. This mechanism of response to low pressure, called pressure-induced vasodilation (PIV), was first described in humans (Fromy et al., 1998). This local neuro-vascular response to low pressure, also found in healthy skin in rats and mice (Fromy et al., 2000; Sigaudo-Roussel et al., 2004), is a physiologically appropriate adjustment of local vasomotor function.

The objective of this review is to give an overview of this cutaneous neuro-vascular interaction and to discuss the changes occurring during aging and diabetes that could put the skin at risk for pressure ulcers.

## The Mechanisms of PIV

**FIGURE 1:**
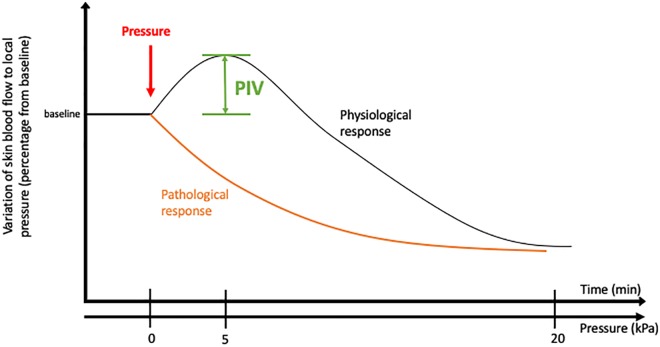
Pressure-induced vasodilatation in healthy and pathological conditions.

To assess PIV in normal conditions, skin blood flow is measured at baseline using a laser Doppler flowmeter. A local external pressure is progressively applied at the surface of the skin, and skin blood flow is measured over time at the local pressure application site. In human adults, the blood flow first increases with increasing pressure until a peak at approximately 5 kPa. Then, the blood flow decreases progressively until the pressure rises to 20 kPa, which marks the end of the PIV phenomenon (Fromy et al., 1998; Figure 1). At the cellular level, the first step of this skin mechanism is the activation of acid-sensing ion channel 3 (ASIC3), a skin mechanoreceptor of sensory C fiber endings (Fromy et al., 2012). Once activated by the local pressure application, these sensory nerve endings release vasodilator neuropeptides, including the calcitonin gene-related peptide (CGRP), vasoactive intestinal peptide (VIP), and/or pituitary adenylate cyclase-activating polypeptide (PACAP). Neurokinins are also released but have no role in PIV development (Fromy et al., 2000; Fizanne et al., 2004). Acute inhibition of nitric oxide synthase (NOS) and cyclooxygenase (COX) totally abolishes PIV (Fromy et al., 2000; Gaubert et al., 2007), suggesting that the main activity pathway of neuropeptides such as CGRP is endothelial dependent. In this case, neuropeptides (CGRP, VIP, and PACAP) will bind to their respective receptors on the endothelial cell surface, causing a sequence of calcium-dependent events via TREK-1 channels (Garry et al., 2007). This calcium-dependent process is crucial in the activation of endothelial NOS (eNOS) and COX, leading to nitric oxide (NO) and prostaglandin (PGI2) production. NO then induces the stimulation of guanylate cyclase in the smooth muscle cell (SMC), resulting in an increase in cyclic guanosine monophosphate (cGMP) (Brain and Grant, 2004), leading to SMC relaxation. Prostaglandins will allow the accumulation of cyclic adenosine monophosphate (AMPc) in endothelial cells by adenylate cyclase activation, which leads to SMC relaxation. Prostaglandins seem to also increase CGRP release by nerve endings and potentiate CGRP vasodilator effects (Holzer et al., 1995). Garry et al. (2005) suggested that the opening of BKCa and KATP channels contributes to the hyperpolarization of vascular SMC to produce PIV development mainly via the NO and prostaglandin pathways, respectively. For now, only potassium channels have been studied in the relaxation mechanism of PIV, but calcium channels might be involved, as in other muscle cells (Zamponi et al., 2015).

**FIGURE 2:**
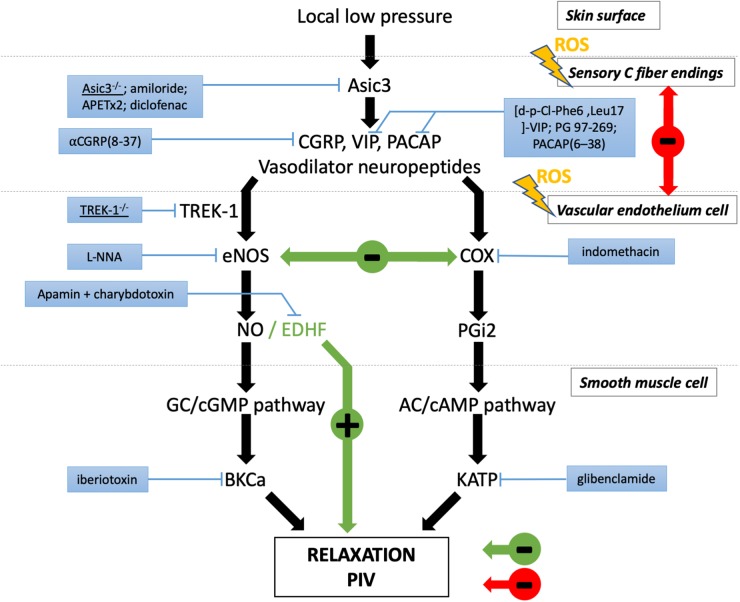
Major players involved in peripheral PIV response to local low pressure. Black arrows represent the physiological pathways of PIV. The effects are indicated in red in diabetes and green in aging. The blue boxes correspond to the pharmacological inhibitors or genetic mutation that revealed the involvement of the designated actor in PIV.

This cascade of events causes vasodilation, allowing skin blood flow to increase from baseline, which delays the decrease in cutaneous blood flow produced by local application of low pressure to the healthy skin, a physiologically appropriate adjustment of local vasomotor function (Figure 2).

## PIV Alteration Associated With Neurovascular Impairment

In some disorders with increased incidence of pressure ulcer, such as aging and diabetes, PIV is altered (Figure 2; Fromy et al., 2010; Vouillarmet et al., 2019). Individuals lacking a normal PIV response show an early decrease in cutaneous blood flow to the application of very low pressures, reflecting a vascular fragility of the skin that increases the risk of ulceration. An interesting point that emerges from the study of microvascular skin alterations in diabetes is the presence of some similarity to physiological aging. The occurrence of peripheral neuropathy in the elderly subject is not something exceptional, estimated at 7% (Hanewinckel et al., 2016).

The degree of PIV alteration in aging and diabetes depends on the severity of the complications, micro-angiopathy and neuropathy, which are common complications. In the absence of peripheral sensory neuropathy, PIV in non-neuropathic older subjects (60–75 years old) is reduced in association with endothelial dysfunction, likely based on an alteration of the NO pathway (Fromy et al., 2010). This age-related endothelial dysfunction is widely reported in humans (Algotsson et al., 1995; Rossi et al., 2002; Tao et al., 2004) and animals (Muller-Delp J.M. et al., 2002; Muller-Delp J. et al., 2002; Woodman and Boujaoude, 2004). To date, several alterations have been found that may partly explain alterations in the vasodilatory function of endothelium cells. First, there is a decrease in NO bioavailability due to a decrease in L-arginine bioavailability (Holowatz et al., 2006), enhanced production of an endogenous inhibitor of NOS (asymmetric dimethylarginine) (Bode-Böger et al., 2005) and tetrahydrobiopterin deficiency (Higashi et al., 2006). There is also a shift in the balance of COX products in favor of vasoconstrictors (thromboxane A2) over vasodilators (prostacyclin PGI2) (El Assar et al., 2012). The appearance of a chronic low-grade inflammatory state called inflamm-aging is associated with increased activity of inducible NOS (iNOS). With aging, there is increased oxidative stress, i.e., increased formation of reactive oxygen species (ROS), especially superoxide. NO produced by iNOS and eNOS can quickly react with superoxide to form peroxynitrite (ONOO-), which leads to oxidative damage in endothelium cells (Cau et al., 2012), vascular relaxation decline and lower ability of the artery to adapt to different flow constraints. At the neuronal level, the increase in ROS induces disturbances in neuronal vascularization, hypoxia, impaired nerve conduction, atrophy, and axonal degeneration (Sifuentes-Franco et al., 2017). Endothelium-derived hyperpolarising factor (EDHF) is merely a back-up system that comes into play under these conditions, as reported in the cutaneous microcirculation in old mice (Gaubert et al., 2007). By partially counteracting the reduction in NO and prostaglandin function, EDHF allows persistent cutaneous PIV in old mice although reduced compared to PIV in young mice. In the absence of peripheral neuropathy, PIV withdrawal observed with aging is due to endothelial dysfunction, as reported in aged mice (Gaubert et al., 2007) and elderly subjects (Fromy et al., 2010). In the presence of peripheral neuropathy in one study, older subjects (60–75 years old) were totally deprived of PIV, leading to early pressure-induced cutaneous ischaemia (Fromy et al., 2010). This inability of the skin to adapt to localized pressure in older subjects was related to the severity of the sensory fiber dysfunction rather than to aging-associated endothelial dysfunction, which was comparable between the non-neuropathic and neuropathic older subjects. Similar findings have been observed in animal studies in diabetic mice exhibiting only vascular dysfunction and diabetic mice exhibiting both vascular and nervous dysfunction (Fromy et al., 2002; Demiot et al., 2006a,b).

In diabetes, the disturbance of the PIV due to either the nervous component (neuropathy) or the vascular component (micro-angiopathy) can be observed in type 2 as well as type 1 diabetes (Koitka et al., 2004; Vouillarmet et al., 2019). The main contributor to vascular and nerve alterations is hyperglycaemia, which persists for many years in type 2 diabetic patients before diagnosis and/or patients who fail to comply with the lifelong treatment. PIV disorders occur rapidly in the presence of hyperglycaemia and persist over time. Hyperglycaemia activates the polyol, protein kinase C, and hexosamine pathways and leads to the accumulation of advanced glycation end products (AGE). Each of these pathways can induce the formation of oxidative stress and the production of ROS a few weeks after hyperglycaemia (Brownlee, 2001).

Recently, Vouillarmet et al. (2019) reported that PIV alteration was more severe in diabetic patients with diabetic foot than in those without. It is interesting to note that the overall neuropathy was not different between groups. However, topical application of lidocaine, a sodium channel blocker used in peripheral neuropathies, did not further decrease PIV in the diabetic foot group, whereas it did so significantly in diabetic patients without diabetic foot. Neuropathy only explains part of the risk of diabetic foot ulcer (DFU), and other parameters can be involved.

Furthermore, type 2 diabetes is often associated with overweight and obesity, which modify microvascular function (de Jongh et al., 2004). Obesity, metabolic dysfunction and type 2 diabetes have been observed in hypercaloric diet (HCD)-fed mice, along with increased dermal adiposity and induced metaflammation (Prattichizzo et al., 2018). Progression of obesity into pre-diabetic and diabetic states is associated with adaptive mechanisms that normalize and then increase the dermal vascular response to pressure (Nguyen-Tu et al., 2019). These adaptive mechanisms are associated with signals whose detection could be a screening tool for overweight and obese patients with or without altered metabolic profiles. Among the several pathological factors induced by hyperglycaemia and affecting the vasculature, the presence of insulin resistance and adiposity could have a key role in maintaining and augmenting the vasodilator capacity of dermal microvessels and thus maintaining PIV. Insulin action on the microvasculature has been suggested to mediate protective effects against diabetes-associated vascular complications (Mikhail and Tuck, 2000) by stimulating Ca^2+^-independent NO production (Hermann et al., 2000) at the onset of insulin resistance that could be impaired later. Since elevated insulin was observed in obese, prediabetic mice along with similar PIV responses to those of age-matched lean mice, hyperinsulinaemia-driven insulin signaling was explored as an actor involved in the adaptive mechanism to normalize PIV. As soon as tissue adiposity increased, there was a decrease in adipokine production and M1-like macrophage recruitment. Adipokines are known to promote vasodilatation and to protect against atherosclerosis; they also facilitate eNOS activity in producing NO and are involved in ROS production mitigation (Fantuzzi, 2005).

These adaptations result in a change in signaling pathways from an NO-dependent response to a pro-inflammatory one, with an increase in COX-2 activity and prostaglandin synthesis. These adaptive mechanisms could explain why in HCD-fed obese, diabetic mice, duration of obesity decreased skin lesions and dermal ischaemia in response to acute skin compression (Nguyen-Tu et al., 2013, 2019; Ozen et al., 2013). However, further studies are needed to inform on whether cyclic skin compression could lead to skin lesions in obese mice.

## Therapeutics to Preserve or Restore PIV in Diabetic Conditions

Different pathways have been studied in mice to understand PIV, and some treatments may improve PIV. The renin-angiotensin system has been implicated in the improvement of neurological and vascular alterations in diabetes (Sifuentes-Franco et al., 2017). In diabetic mice with neuropathy deprived of PIV, the use of candesartan, an angiotensin II type 1 receptor blocker (ARB), restored the PIV and thus the endothelium-dependent response but also restored the normal nociceptive threshold (Danigo et al., 2015; Sifuentes-Franco et al., 2017). Currently, there is no clinical trial on human diabetic patients to assess the effect of ARB on the restoration of the PIV or to treat pressure ulcer, where the PIV could be involved. Margolis et al. (2010) carried out a retrospective study in the United Kingdom on 40,342 diabetic patients in 2010. They looked at the differential effect of ARB and angiotensin-converting enzyme inhibitors (ACEi), notably on DFU. They showed that diabetic patients on ARB had a lower risk for developing DFU than those on ACEi (hazard ratio (HR) 0.50 95% CI [0.43–0.59]). Most interestingly, diabetic patients on ARB had a lower risk of developing DFU (HR 0.84 [0.78–0.91]) than those without any treatment. This difference was not observed with ACEi. In this retrospective study, ARB seemed to prevent the formation of DFU in diabetic patients. It is not known how ARB prevented DFU in those patients, and it is impossible to confirm the role of a restoration of PIV by ARB use. Nevertheless, it seems to be a promising avenue of research to explore PIV and pressure ulcer, especially in diabetes. Another path that has been explored is oxidative stress. Hyperglycaemia occurring during uncontrolled diabetes increases ROS, one of the main pathways of damage leading to the formation of AGE. The disappearance of PIV after 1 week of hyperglycaemia in mice suggested the rapid damage done by ROS to the endothelium and the importance of this mechanism in PIV alteration. Demiot et al. (2006a,b) studied three treatments sorbinil, alagebrium, and alpha-lipoic acid acting at three different levels of the oxidative pathways for their ability to restore PIV. Diabetes was induced in mice by streptozotocin, and after 1 week of hyperglycaemia, only vascular damage appeared, whereas at 8 weeks of diabetes, mice exhibited vascular dysfunction and peripheral neuropathy. Sorbinil, an aldose reductase inhibitor, did not allow restoration of the endothelium-dependent response or PIV in short-term-diabetic mice. In contrast, in long-term-diabetic mice exhibiting neuropathy, 2-week sorbinil treatment was able to restore both the endothelium-dependent response and PIV. The nerve response was only improved for the thermal nociceptive response, and there was no effect on the motor nerve conduction velocity. Alagebrium treatment, an AGE-crosslink breaker, had no effect on the endothelium-dependent response or PIV in short-term-diabetic mice. At 8 weeks of diabetes, 2-week alagebrium treatment permitted the restoration of the endothelium-dependent response, while PIV was only improved but not completely restored, with no improvement of the nerve response. Alpha-lipoic acid (LPA) has antioxidant properties and was only tested in short-term diabetes, where it showed a complete restoration of both the endothelium-dependent response and PIV. Of these three treatments, LPA was the only one to restore PIV at an early stage of diabetes. Sorbinil, thanks to its action on nervous- and endothelium-dependent responses, restored PIV at a late stage of diabetes with neuropathy. Unfortunately, the ability to prevent pressure ulcer was not investigated, nor was the persistence of PIV improvement.

## Perspectives for Patients at Risk for Pressure Ulcer

These results are encouraging, suggesting further investigations, especially clinical prospective studies. Mechanisms leading to this PIV alteration are different according to the type of diabetes, but in both cases the alteration results from the dysregulation of glucose homeostasis. In type 2 diabetes, there is chronic hyperglycaemia, microvascular damage with or without neuropathy and obesity in a significant part of the population. Screening for earlier microvascular damage and subclinical neuropathy is possible thanks to the ability to measure PIV using a non-invasive skin laser Doppler flowmeter. This appears to be an interesting way to detect microvascular damage and incipient neurological alteration, even more since a link has been established between PIV alteration and DFU (Vouillarmet et al., 2019). Indeed, the vasodilator capacity in response to pressure was significantly lower in patients with DFU compared to those without DFU, suggesting that PIV alteration is a good marker of skin vulnerability and could be used to better predict individuals at risk. The life-time prevalence of DFU (10–20%) and the associated consequences (infection, amputation, lower quality of life, etc.) make it an important healthcare issue (Margolis et al., 2010).

For type 1 diabetes, no clinical trial on PIV has been conducted to date. Nevertheless, given the high incidence of microangiopathy in relation to chronicity of diabetes (Katsarou et al., 2017) and rapid onset of PIV alteration in hyperglycaemia (Sigaudo-Roussel et al., 2004), screening for microvascular alterations through the study of PIV (Fromy et al., 2012; Vouillarmet et al., 2019) appears to be a promising avenue for research.

In aging, PIV exists in non-neuropathic older adults but is totally absent in old subjects with sensory peripheral neuropathy (Fromy et al., 2010). Accordingly, sensory neuropathy is a risk factor for pressure ulcers, particularly in individuals with diabetes mellitus (Boulton et al., 2004), and sensory perception is a criterion used for predicting pressure ulcer risk in hospitalized individuals (Benoit and Mion, 2012). In a cross-sectional study including 210 older hospitalized adults (mean age 85), Gaubert-Dahan et al. (2013) reported that the severity of sensory peripheral neuropathy according to the neuropathy symptom score was highly associated with the prevalence and severity of heel pressure ulcer. Indeed, the heel pressure ulcer prevalence was 4, 11, and 26% in older adults with light, moderate, and severe sensory neuropathy, respectively. Sensory peripheral neuropathy appears to be the most critical factor, emphasizing the crucial role of sensory nerves in the physiological protection against pressure ulcer. Altogether, these studies show that impaired PIV indicates early skin frailty and provide new insights into pressure ulcer prevention in elderly and diabetic patients.

## Conclusion

Pressure-induced vasodilation is an important mechanism of cutaneous homeostasis in response to low pressure. Its alteration in aging and diabetes is a reflection of nervous- and/or endothelium-dependent response damage due to hyperglycaemia and oxidation. In diabetes, insulin and adiposity can influence PIV over time. In overweight and obese subjects with or without metabolic disease, highlighting specific vasodilatory signals may help to assess pressure ulcer risk. The ARBs and, notably, candesartan are an interesting way to restore PIV and heal pressure ulcer for diabetic patients. Interestingly, PIV alterations in diabetes share some common points with PIV alterations in aging. Inflamm-aging will lead to oxidative stress and cells damages on vascular endothelium and nerves during aging, leading to PIV alteration.

Finally, assessment of PIV in elderly subjects and diabetic patients could be an interesting screening tool to identify microvascular and neurological alterations without clinical symptoms and delay the appearance of pressure ulcer.

Further studies on the roles of different neurotransmitters are needed to develop new treatments to improve PIV and help to prevent pressure ulcer in these pathological conditions.

## Author Contributions

MF wrote the first draft of the manuscript. All authors contributed to the manuscript revision, read, and approved the submitted version.

## Conflict of Interest Statement

The authors declare that the research was conducted in the absence of any commercial or financial relationships that could be construed as a potential conflict of interest.

## Abbreviations

AC: adenylate cyclase
AMPc: cyclic adenosine monophosphate
ASIC3: acid-sensing ion channel 3
BKCa: large conductance calcium- and voltage-activated potassium channel
cGMP: cyclic guanosine monophosphate
CGRP: calcitonin gene-related peptide
COX: cyclooxygenase
EDHF: endothelium-derived hyperpolarising factor
eNOS: endothelial nitric oxide synthase
GC: guanylate cyclase
KATP: ATP-sensitive potassium
NO: nitric oxide
PACAP: pituitary adenylate cyclase-activating polypeptide
PGi2: prostacyclin
ROS: reactive oxygen species
VIP: vasoactive intestinal peptide.
